# Supplementary material for: Porous borders at the wild-crop interface promote weed adaptation in Southeast Asia
Source: Nat Commun. 2024 Feb 21;15:1182. doi: 10.1038/s41467-024-45447-0 (PMC10881511; doi:10.1038/s41467-024-45447-0)
Supplement: Supplementary file 1 — Supplementary Information [file 41467_2024_45447_MOESM1_ESM.pdf]

Porous borders at the wild-crop interface promote weed adaptation in Southeast Asia

Li *et al*.

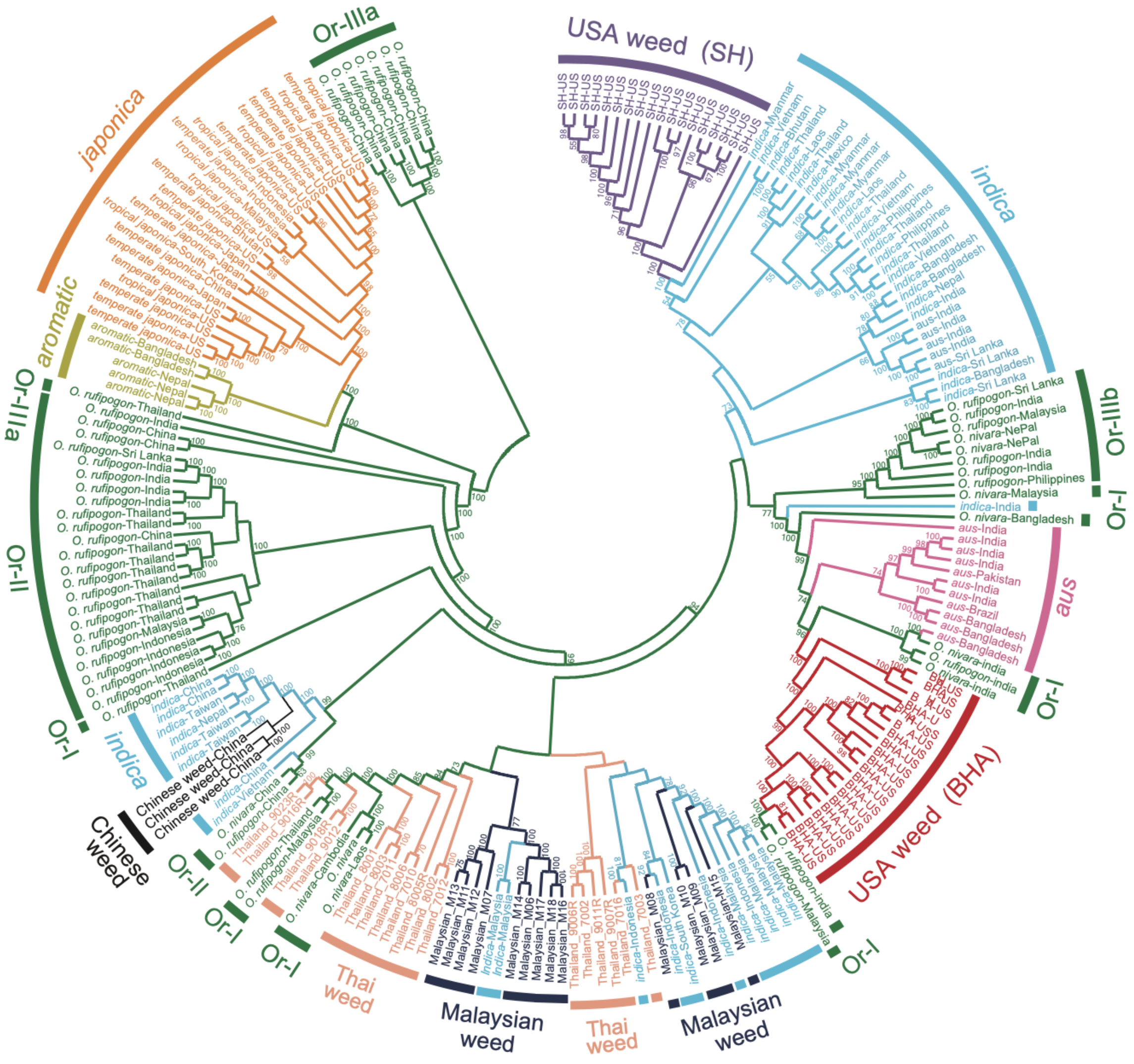

Supplementary Figure 1. Neighbor-Joining tree of the 217 worldwide rice accessions. Colors represent different rice groups, and numbers above each branch indicate bootstrap support values (>50%). Accession labels indicate geographic origin. Tree topologies were rooted using wild rice Or-IIIa group as the outgroup according to Huang *et al*.[1] and Li *et al*.[2]. Source data are provided as a Source Data file.

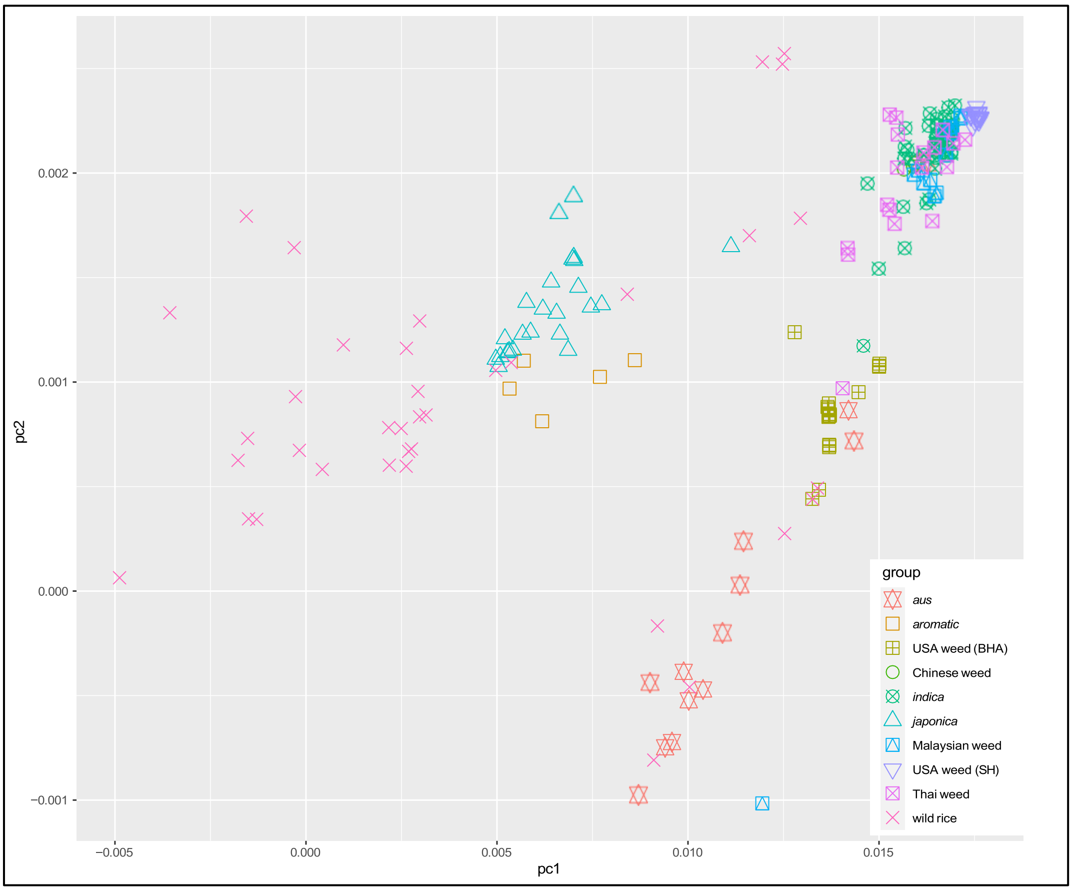

Supplementary Figure 2. Principal component analysis (PCA) of the 217 global rice accessions based on whole genome SNP dataset. Each rice group is shown by different colored symbols. Source data are provided as a Source Data file.

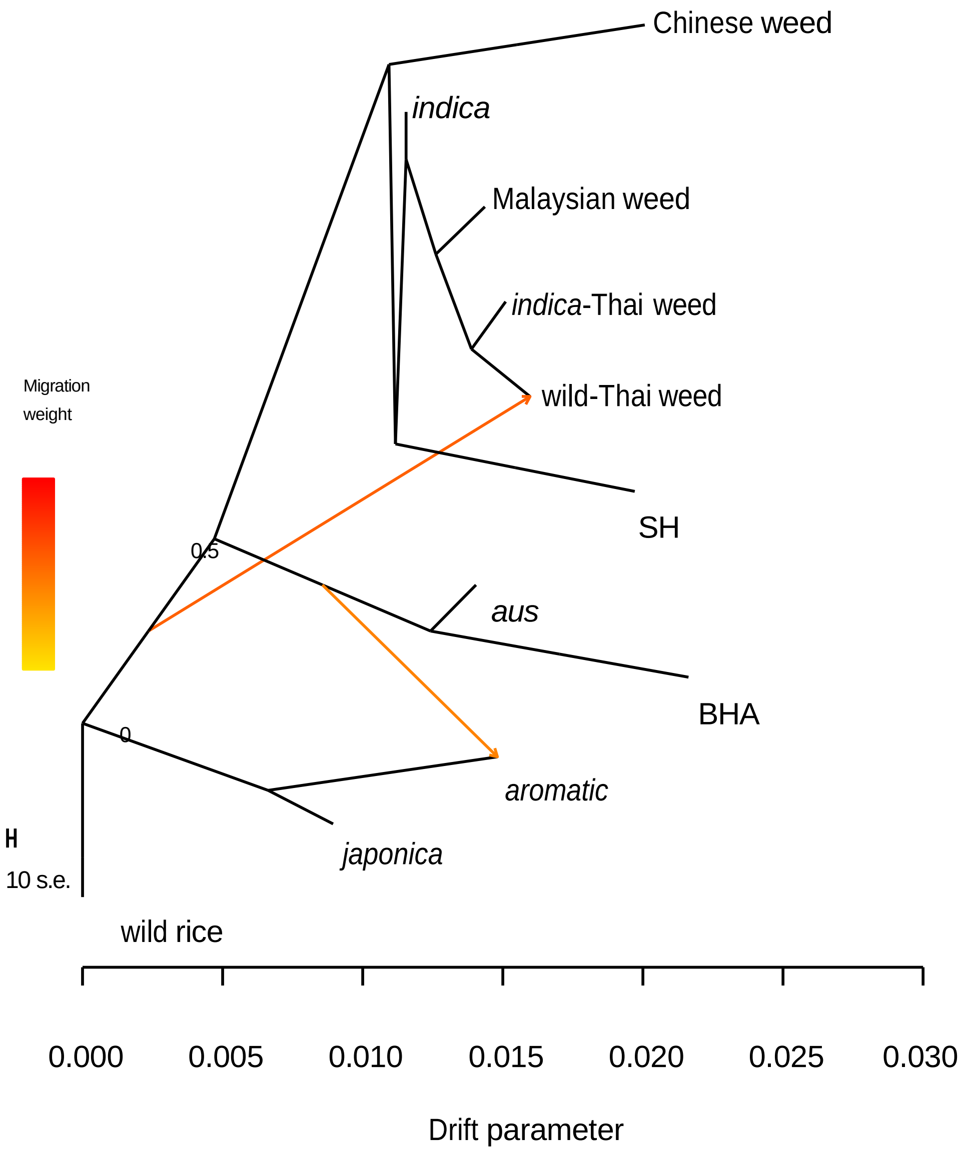

Supplementary Figure 3. Direction and level of gene flow detected among the wild, cultivated and weedy rice groups. Yellow to red color gradient represents low to high levels of inferred gene flow. The bar on the bottom indicates the drift parameter. Estimate of gene flows among the 11 groups is analyzed based on VCF file that contained all variant information of the 217 rice accessions.

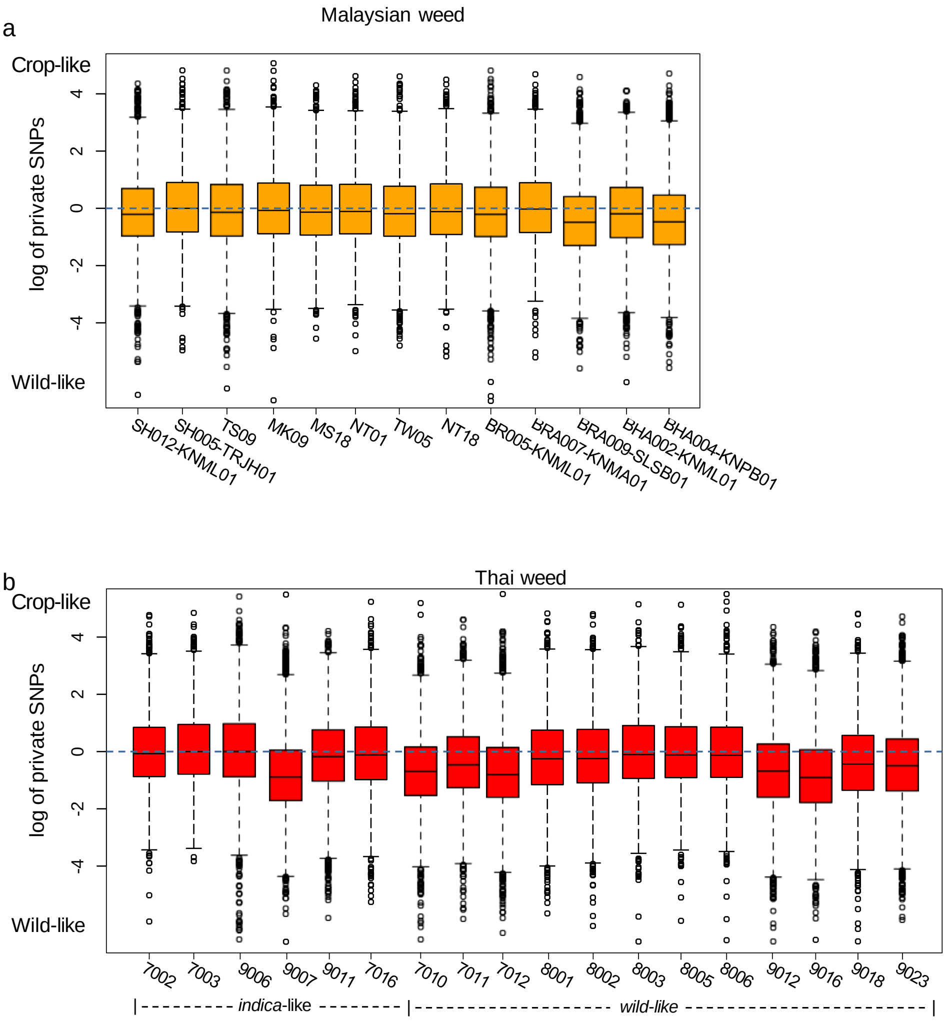

Supplementary Figure 4. Ratio of crop- and wild-specific private SNPs identified in individual Malaysian and Thai weedy accessions. The blue dashed line indicates an equal number of crop- and wild-specific private SNPs within the 100-kb sliding window. The box indicates 95% of the ratio values between crop-specific and wild-specific private SNPs in each of these weedy rice strains. The line within each box indicates the median value for each weedy rice strain. The numbers of private SNPs are included in Supplementary Data 4 and 5. Accession numbers of these weedy rice strains are shown in Supplementary Data 1. Source data are provided as a Source Data file.

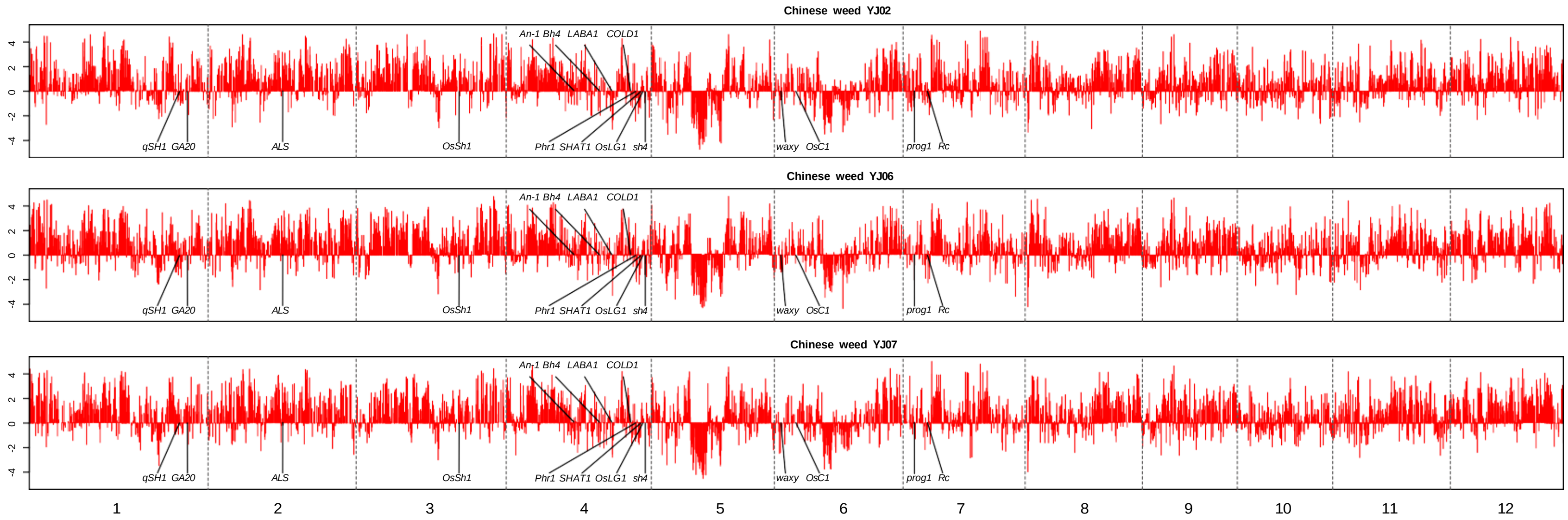

Supplementary Figure 5. **Distribution of crop- and wild-specific private SNPs across the 12 rice chromosomes for Chinese weedy rice samples.** Chromosome numbers are indicated along the x axis. Positive values are differentially crop-like, and negative values are differentially wild-like. Each row indicates a single weedy accession. Black arrows indicate the chromosomal locations of major domestication or crop improvement genes. Source data are provided as a Source Data file.

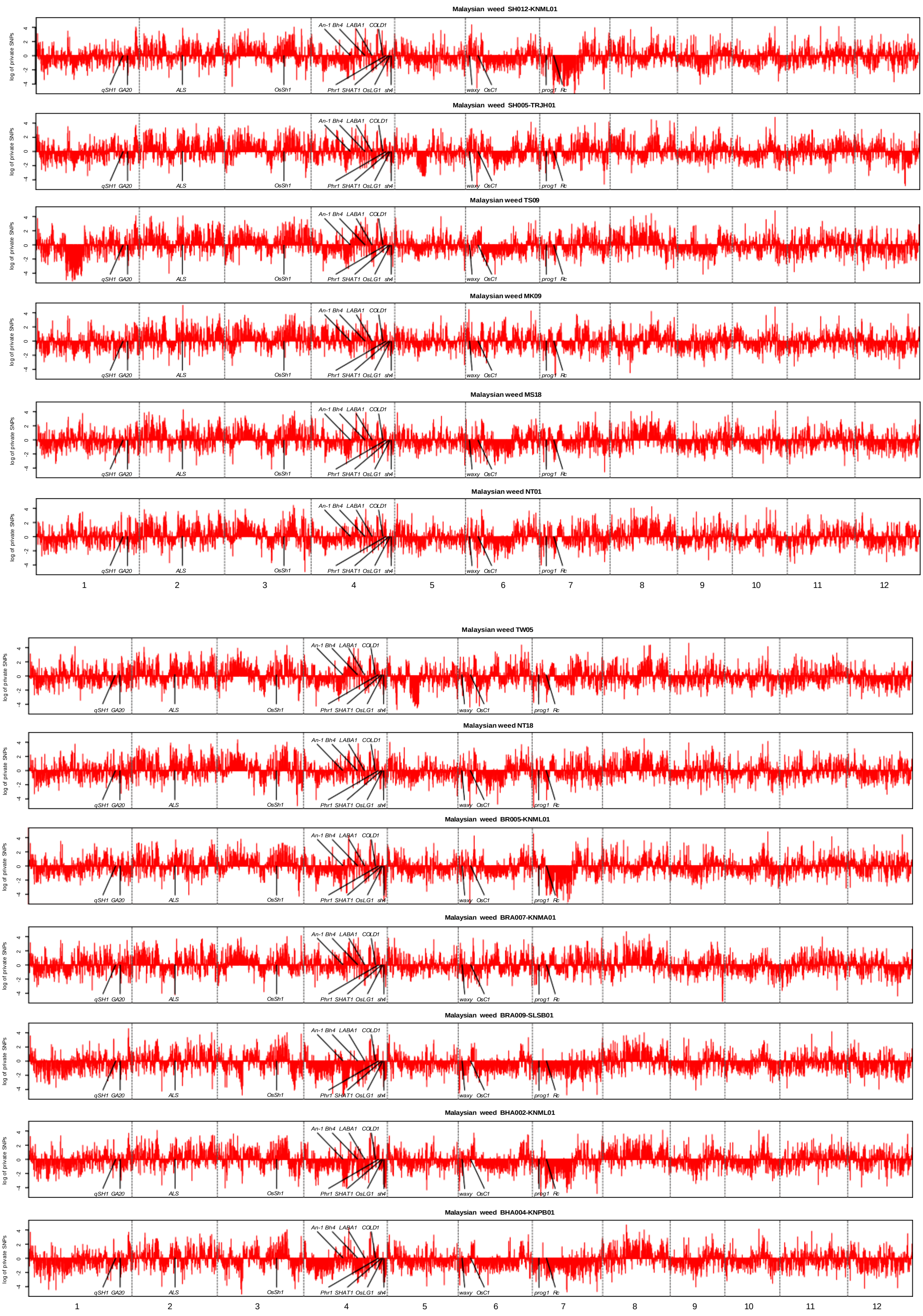

Supplementary Figure 6. Distribution of crop- and wild-specific private SNPs across the 12 rice chromosomes for Malaysian weedy rice samples. Chromosome numbers are indicated along the x axis. Positive values are differentially crop-like, and negative values are differentially wild-like. Each row indicates a single weedy accession. Black arrows indicate the chromosomal locations of major domestication or crop improvement genes. Source data are provided as a Source Data file.

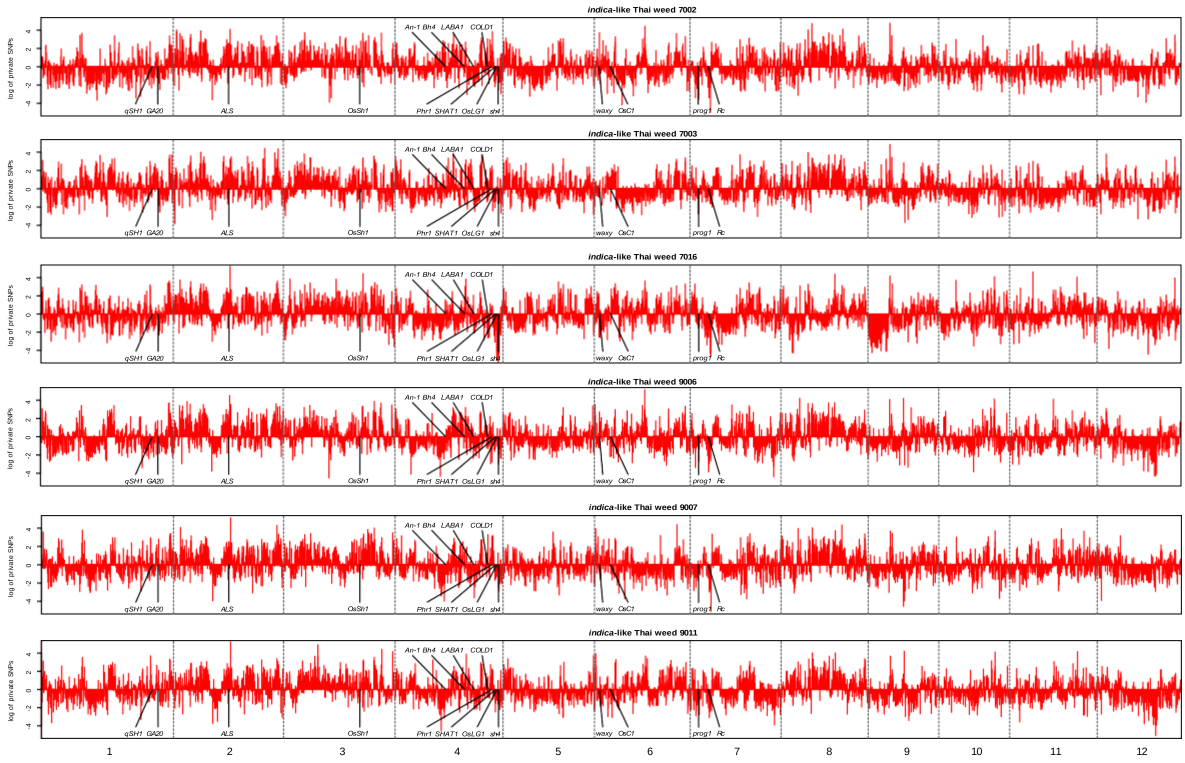

Supplementary Figure 7. **Distribution of crop- and wild-specific private SNPs across the 12 rice chromosomes for *indica*-like Thai weedy rice samples.** Chromosome numbers are indicated along the x axis. Positive values are differentially crop-like, and negative values are differentially wild-like. Each row indicates a single weedy accession. Black arrows indicate the chromosomal locations of major domestication or crop improvement genes. Source data are provided as a Source Data file.

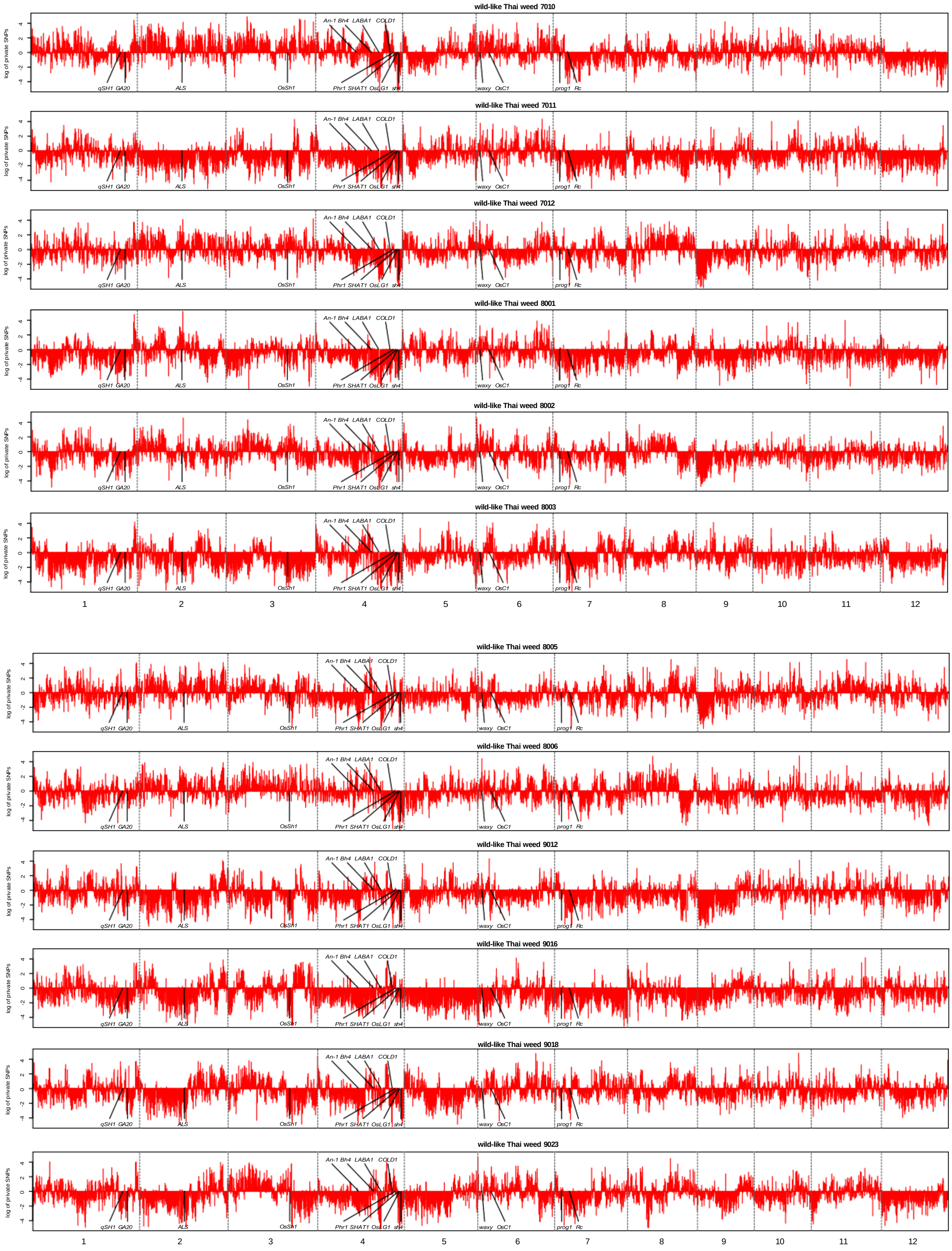

Supplementary Figure 8. **Distribution of crop- and wild-specific private SNPs across the 12 rice chromosomes for wild-like Thai weedy weedy rice samples.** Chromosome numbers are indicated along the x axis. Positive values are differentially crop-like, and negative values are differentially wild-like. Each row indicates a single weedy accession. Black arrows indicate the chromosomal locations of major domestication or crop improvement genes. Source data are provided as a Source Data file.

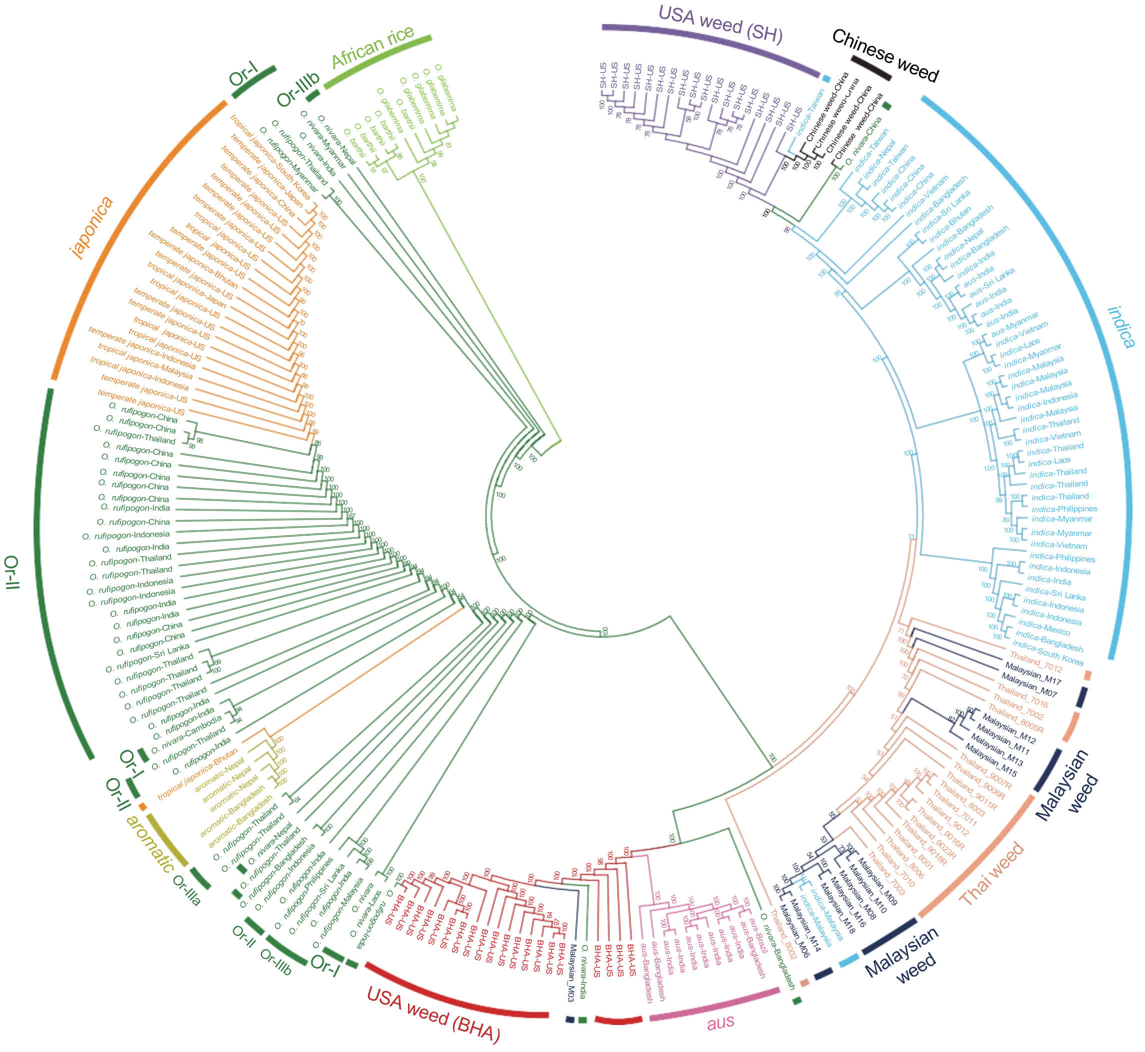

Supplementary Figure 9. Neighbor-Joining tree of the 217 worldwide Asian rice accessions and 10 African rice accessions. Colors represent different rice groups, and numbers above each branch indicate bootstrap support values (>50%). Accession labels indicate geographic origin. Tree topologies were rooted using *Oryza barthii* and *Oryza glaberrima*. Source data are provided as a Source Data file.

Supplementary Table 1. Cross validation value for 217 rice accessions.

K	Cross validation value
1	0.18072
2	0.16394
3	0.15224
4	0.1488
5	0.14719
6	0.14325
7	0.14484
8	0.15009
9	0.1416
10	0.15143

Supplementary references

Huang, X. *et al*. A map of rice genome variation reveals the origin of cultivated rice. *Nature* **490**, 497-501 (2012).

Li, L.F., Li, Y.L., Jia, Y., Caicedo, A.L. & Olsen, K.M. Signatures of adaptation in the weedy rice genome. *Nat. Genet*. **49**, 811-814 (2017).
