# Supplementary material for: Porous borders at the wild-crop interface promote weed adaptation in Southeast Asia
Source: Nat Commun. 2024 Feb 21;15:1182. doi: 10.1038/s41467-024-45447-0 (PMC10881511; doi:10.1038/s41467-024-45447-0)
Supplement: Supplementary file 3 — Description of Additional Supplementary Files [file 41467_2024_45447_MOESM3_ESM.pdf]

Description of Additional Supplementary Files

File Name: Supplementary Data 1
Description: The list of 217 accessions of wild, cultivated and weedy rice species sampled in the collection.

File Name: Supplementary Data 2
Description: Genetic assignments and heterozygosity for each wild, cultivated and weedy rice accession.

File Name: Supplementary Data 3
Description: Nucleotide diversity (π) and number of raw variants (SNPs and INDELs) for each chromosome of wild, cultivated and weedy rice.

File Name: Supplementary Data 4
Description: Number and proportion of wild- and crop-specific private SNPs in Asian weedy rice strains for each chromosome.

File Name: Supplementary Data 5
Description: Number and proportion of wild- and crop-specific private SNPs in each weedy rice strain for each chromosome.

File Name: Supplementary Data 6
Description: Causative mutations of the domestication genes in the 34 Malaysian and Thai weedy accesssions.

File Name: Supplementary Data 7
Description: Nucleotide diversity (π and θ) and genetic differentiation (Fst) of the candidate genomic regions in Malaysian weed compared to indica.

File Name: Supplementary Data 8
Description: Nucleotide diversity (π and θ) and genetic differentiation (Fst) of the candidate genomic regions in indica-like Thai weed compared to indica.

File Name: Supplementary Data 9
Description: Nucleotide diversity (π and θ) and genetic differentiation (Fst) of the candidate genomic regions in wild-like Thai weed compared to indica.

File Name: Supplementary Data 10
Description: Top 1% of the candidate genomic regions showed selective sweep in the three types of SEA weedy rice.

File Name: Supplementary Data 11
Description: Genomic regions of shared low nucleotide diversity (π) among the analyzed weedy strains.
