# Supplementary material for: Porous borders at the wild-crop interface promote weed adaptation in Southeast Asia
Source: Nat Commun. 2024 Feb 21;15:1182. doi: 10.1038/s41467-024-45447-0 (PMC10881511; doi:10.1038/s41467-024-45447-0)
Supplement: Supplementary file 15 — Reporting Summary [file 41467_2024_45447_MOESM15_ESM.pdf]

nature portfolio

Corresponding author(s): Kenneth M. Olsen
Beng-Kah Song

Last updated by author(s): Jan 22, 2024

Reporting Summary

Nature Portfolio wishes to improve the reproducibility of the work that we publish. This form provides structure for consistency and transparency in reporting. For further information on Nature Portfolio policies, see our Editorial Policies and the Editorial Policy Checklist.

Statistics

For all statistical analyses, confirm that the following items are present in the figure legend, table legend, main text, or Methods section.

n/a	Confirmed	
☐	☒	The exact sample size (*n*) for each experimental group/condition, given as a discrete number and unit of measurement
☐	☒	A statement on whether measurements were taken from distinct samples or whether the same sample was measured repeatedly
☒	☐	The statistical test(s) used AND whether they are one- or two-sided<br>*Only common tests should be described solely by name; describe more complex techniques in the Methods section.*
☐	☒	A description of all covariates tested
☐	☒	A description of any assumptions or corrections, such as tests of normality and adjustment for multiple comparisons
☐	☒	A full description of the statistical parameters including central tendency (e.g. means) or other basic estimates (e.g. regression coefficient) AND variation (e.g. standard deviation) or associated estimates of uncertainty (e.g. confidence intervals)
☒	☐	For null hypothesis testing, the test statistic (e.g. *F*, *t*, *r*) with confidence intervals, effect sizes, degrees of freedom and *P* value noted<br>*Give P values as exact values whenever suitable.*
☐	☒	For Bayesian analysis, information on the choice of priors and Markov chain Monte Carlo settings
☐	☒	For hierarchical and complex designs, identification of the appropriate level for tests and full reporting of outcomes
☒	☐	Estimates of effect sizes (e.g. Cohen's *d*, Pearson's *r*), indicating how they were calculated

Our web collection on statistics for biologists contains articles on many of the points above.

Software and code

Policy information about availability of computer code

Data collection	Clean reads were mapped onto the Nipponbare reference genome (temperate japonica, MSU 6.0 version, http://rice.plantbiology.msu.edu) using BWA (Li and Durbin, 2009) with the parameter “bwa aln -n 0.05”. Raw variants (insertions and deletions (INDELs) and SNPs) were then realigned with the Genome Analysis Toolkit (GATK) IndelRealigner version 2.6 (McKenna et al., 2010). Genotype calling of the realigned assemblies was performed using SAMtools (Li et al., 2010). VCF matrices that were generated from Asian and African rice accessions were combined as an integrated dataset using VCFtools (Danecek et al., 2011).<br>References:<br>Li, H. & Durbin, R. Fast and accurate short read alignment with Burrows–Wheeler transform. Bioinformatics 25, 1754-1760 (2009).<br>McKenna, A. et al. The Genome Analysis Toolkit: a MapReduce framework for analyzing next-generation DNA sequencing data. Genome Res. 20, 1297-1303 (2010).<br>Li, H. et al. The sequence alignment/map format and SAMtools. Bioinformatics 25, 2078-2079 (2009).<br>Danecek, P. et al. The variant call format and VCFtools. Bioinformatics 27, 2156- 2158(2011).
Data analysis	MEGA7 {Kumar et al. 2016}; ADMIXTURE {Alexander et al., 2009}; Treemix (Pickrell et al., 2012); VCFtools {Danecek et al., 2011}; SweeD {Pavlidis et al., 2013}<br>References:<br>Kumar S, Stecher G, Tamura K. {2016) MEGA7: Molecular evolutionary genetics analysis version 7.0 for bigger datasets. Molecular Biology and Evolution 33, 1870-1874.<br>Alexander DH, Novembre J, Lange K. {2009) Fast model-based estimation of ancestry in unrelated individuals. Genome Research 19,1655-1664.<br>Pickrell JK and Pritchard JK. {2012) Inference of population splits and mixtures from genome-wide allele frequency data. PLOS Genetics 8,

el002967.
Danecek P, Auton,_'7-A, becasis G, et al. {2011} The variant call format and VCFtools. Bioinformatics 27, 2156-2158.
Pavlidis, P., Živković, D., Stamatakis, A. & Alachiotis, N. SweeD: likelihood-based detection of selective sweeps in thousands of genomes. Mol. Biol. Evol. 30, 2224-2234 (2013).

For manuscripts utilizing custom algorithms or software that are central to the research but not yet described in published literature, software must be made available to editors and reviewers. We strongly encourage code deposition in a community repository (e.g. GitHub). See the Nature Portfolio guidelines for submitting code & software for further information.

Data

Policy information about availability of data

All manuscripts must include a data availability statement. This statement should provide the following information, where applicable:

- Accession codes, unique identifiers, or web links for publicly available datasets
- A description of any restrictions on data availability
- For clinical datasets or third party data, please ensure that the statement adheres to our policy

The raw total sequence reads have been deposited into Genome Warehouse in National Genomics Data Center, Chinese Academy of Sciences/China National Center for Bioinformation, under the project number PRJCA016178 (https://www.cncb.ac.cn/search/specific?dbId=bioproject&q=PRJCA016178)

Research involving human participants, their data, or biological material

Policy information about studies with human participants or human data. See also policy information about sex, gender (identity/presentation), and sexual orientation and race, ethnicity and racism.

Reporting on sex and gender	N/A
Reporting on race, ethnicity, or other socially relevant groupings	N/A
Population characteristics	N/A
Recruitment	N/A
Ethics oversight	N/A

Note that full information on the approval of the study protocol must also be provided in the manuscript.

Field-specific reporting

Please select the one below that is the best fit for your research. If you are not sure, read the appropriate sections before making your selection.

☐ Life sciences ☐ Behavioural & social sciences ☒ Ecological, evolutionary & environmental sciences

For a reference copy of the document with all sections, see nature.com/documents/nr-reporting-summary-flat.pdf

Ecological, evolutionary & environmental sciences study design

All studies must disclose on these points even when the disclosure is negative.

Study description	This study used whole genome resequencing data to compare the evolution of Southeast Asian weedy rice to weedy, cultivated and wild rice found in sympatry and other regions worldwide. Quantitative measurements are not part of the experimental design.
Research sample	Whole genome sequences of 217 wild, weedy and cultivated rice samples. Thirty-four Southeast Asian weedy and cultivated rice accessions, including 31 weedy rice strains from Thailand and Malaysia, were compared to published genome sequences of 183 other samples worldwide, encompassing wild, cultivated and weedy rice strains. Weedy rice accessions were selected to represent Southeast Asian weedy rice based on their genetic background and morphological traits as assessed in previous studies.
Sampling strategy	Sampling in Southeast Asia was designed to maximize areas of sympatry with wild rice. Sampling numbers were determined by combined criteria of geographical representation of the focal region and costs of generating high-quality genome sequence assemblies for analysis. ll sequenced weedy rice accessions were derived from either mature plants or seeds on maturing inflorescences that were collected within rice fields in regions of weedy rice infestations. Collecting was performed with the permission of local farmers. Sampling was deemed sufficient when it satisfied the dual criteria of geographical representation and high-quality genome sequence data for the selected accessions.
Data collection	Data were obtained from whole genome sequences following raw data processing, alignment and filtering to yield reliable genome-wide SNP data. Methods for data collection are described in the Software and Code section above. Co-contributing authors performed these steps. Data collection was performed by LFL, MJW, BKS and YH using raw Illumina sequence read files and derived alignments.

Timing and spatial scale	All samples are contemporary and reflect recent population dynamics in Oryza species. Spatial scale is described above.
Data exclusions	No data were excluded other than low-quality SNP calls that were filtered prior to data analysis.
Reproducibility	The study does not involve experimental manipulations and controls. The large sample sizes and global geographical representation supports the likelihood of reproducibility of key inferences.
Randomization	Accessions were assigned to genetic subgroups based on phylogenetic distance trees and Bayesian analyses to assess genetic structure. Sampling was designed to have roughly equal sample sizes among genetic groups.
Blinding	Population structure analyses were performed blind with respect to labeling in germ plasm collections.

Did the study involve field work? [x] Yes [ ] No

Field work, collection and transport

Field conditions	Rice fields in Malaysia and Thailand
Location	Sampling origins are provided for all accessions in the Supplementary Information (Supplementary Data 1).
Access & import/export	Sampling occurred in agricultural fields with the permission of local farmers.
Disturbance	N/A

Reporting for specific materials, systems and methods

We require information from authors about some types of materials, experimental systems and methods used in many studies. Here, indicate whether each material, system or method listed is relevant to your study. If you are not sure if a list item applies to your research, read the appropriate section before selecting a response.

Materials & experimental systems

n/a	Involved in the study	
[x]	[ ]	Antibodies
[x]	[ ]	Eukaryotic cell lines
[x]	[ ]	Palaeontology and archaeology
[x]	[ ]	Animals and other organisms
[x]	[ ]	Clinical data
[x]	[ ]	Dual use research of concern
[ ]	[x]	Plants

Methods

n/a	Involved in the study	
[x]	[ ]	ChIP-seq
[x]	[ ]	Flow cytometry
[x]	[ ]	MRI-based neuroimaging

Dual use research of concern

Policy information about dual use research of concern

Hazards

Could the accidental, deliberate or reckless misuse of agents or technologies generated in the work, or the application of information presented in the manuscript, pose a threat to:

No	Yes	
☒	☐	Public health
☒	☐	National security
☒	☐	Crops and/or livestock
☒	☐	Ecosystems
☒	☐	Any other significant area

Experiments of concern

Does the work involve any of these experiments of concern:

No	Yes	
☒	☐	Demonstrate how to render a vaccine ineffective
☒	☐	Confer resistance to therapeutically useful antibiotics or antiviral agents
☒	☐	Enhance the virulence of a pathogen or render a nonpathogen virulent
☒	☐	Increase transmissibility of a pathogen
☒	☐	Alter the host range of a pathogen
☒	☐	Enable evasion of diagnostic/detection modalities
☒	☐	Enable the weaponization of a biological agent or toxin
☒	☐	Any other potentially harmful combination of experiments and agents

Plants

Seed stocks	Plant material for whole genome sequencing was obtained as described above under Sampling strategy and Data collection.
Novel plant genotypes	N/A
Authentication	N/A
